# A prospective survey on trephine biopsy of bone and bone marrow: an experience with 274 Indian patients’ biopsies

**DOI:** 10.1186/s40001-023-01167-7

**Published:** 2023-06-24

**Authors:** Rahul Nadda, Ramjee Repaka, Nabhajit Mallik, Ashish Kumar Sahani

**Affiliations:** 1 Department of Biomedical Engineering, Indian Institute of Technology Ropar, Rupnagar, Punjab 140001 India; 2 Department of Mechanical Engineering, Indian Institute of Technology Ropar, Rupnagar, Punjab 140001 India; 3 Department of Hematology, Postgraduate Institute of Medical Education and Research, Chandigarh, 160012 India

**Keywords:** Bone marrow, Trephine biopsy, Survey, Hematology, Clinical analysis, Pain

## Abstract

Trephine bone marrow biopsy is an effective technique for diagnosing hematological malignancies in patients of different ages. During trephine biopsy, bone marrow cores are obtained for detailed morphological evaluation to look for any abnormality and arrive at a diagnosis. The primary goal of this work is to perform a survey on Indian patients of various ages for the trephine bone marrow biopsy process. In the present study, data related to 274 trephine biopsy samples from 300 patients were acquired at the Post Graduate Institute of Medical Education and Research (PGIMER) in Chandigarh, India. Pain was found to be the sole major procedure-related complication, and patients reported no/less pain in 41 BMB (14.96%) patients, moderate pain in 82 (29.92%) cases, and unbearable pain in 151 (55.1%) BMB cases. In addition, the patients were evaluated by the authors and hematologist as non-anxious for the procedure in 34 (12.4%), anxious in 92 (33.57%), and very/highly anxious in 148 (56%) cases. The bone texture of the patients significantly affected the needle bending, number of repetitions required, and size of the bone marrow sample. This demonstrates the need for improvement in the biopsy procedure. To this end, a survey was conducted to assess the numerous difficulties and diagnostic outcomes throughout the trephine biopsy process.

## Introduction

The trephine bone marrow biopsy is a significant medical procedure for diagnosing various hematological and non-hematological disorders. It is also essential for the follow-up inspection of patients who have undergone medical treatments, such as chemotherapy and bone marrow transplantation. Palpation-assisted techniques are generally used to perform this procedure. The said procedures have a minimal complication rate and high accuracy [1]. In 1903 Pianese first described bone marrow sampling for pathological analysis using trephination of the femur [2–4]. Many researchers were discouraged from collecting bone marrow samples due to the paucity of active bone marrow in the tibia, but then in the 1920s, Arinkin and Seyfarth revealed the use of sternal needle penetration as an ideal approach for acquiring clinical bone marrow aspirate samples [4, 5]. In 1930 Custer [6] demonstrated the clinical significance of biopsy sample preparations and histologic segmentation of sternal bone with smear preparations. The author used this approach to remove a 1 cm diameter disc from the ventral sternal plate, which required a difficult intra-operative procedure. However, before 1958, only a small number of bone marrow biopsies (BMB) were carried out. At this time, McFarland and Dameshek [7] outlined a more typical approach using the Vim-Silverman BMB needle. Subsequently, Schadt Fischer, Jensen, Westerman, Jamshidi, and others improved the Vim-Silverman needle, increasing biopsy retention, making the technique less unpleasant, and providing a large core sample [8–11]. Trephine needles have lately experienced engineering improvements, including precisely built cannula tips and stylets. This allows for smooth cortical bone penetration and the deployment of an inner core capture device to separate the sample and prevent core damage during needle extraction [12, 13].

A bone marrow evaluation is critical to analyzing patients suffering from a wide range of hematological and non-hematological problems and is essential for their diagnosing, staging, follow-up, and prognosis [14]. Trephine biopsy involves obtaining a sample from the posterior iliac crest (most commonly) using a Jamshidi or other comparable needle and handling it in the same manner as other surgical samples. Trephine biopsy can be performed with minimal discomfort to the patient if appropriate local anesthesia is provided. According to a 10-year-old English national study, it is a safe procedure with only 0.07% of adverse incidences [1]. Infection, discomfort, and bleeding are major consequences of posterior iliac crest bone marrow procedures, while cardiac tamponade following sternal trephine biopsy is exceedingly rare. Bleeding in trephine bone marrow biopsy is considered the typical and most harmful severe event, but pain evaluation and its control are poorly addressed [15–19]. Modern advances in marrow-procurement technology and trephine bone marrow biopsy needles with specially designed cutting edges have shortened procedure time and enhanced sample quality. In most nations, the posterior iliac crest is the preferred site for a trephine bone marrow biopsy [20]. The sternal manubrium is more accessible than the iliac crest, especially in elderly, very fatty, or difficult-to-move individuals, and is very useful for bone marrow aspiration. It enables pressure on the wound to maintain adequate hemostasis [14]. Whatever the biopsy location, trephine bone marrow biopsy is still expected to be a painful procedure. Most information on pain evaluation in adults or children was from patients who had often undergone BMA followed by trephine bone marrow biopsy [21–23]. Before performing a trephine bone marrow biopsy, several approaches for anesthesia have been suggested, but no consensus has yet been reached [20]. Pain is associated with various sociodemographic factors, such as age, gender, level of education, ethnicity, body mass index, and physical and other psychological factors. Biopsy procedure, including the type of needles used for biopsy, also plays a role [22]. Extreme anxiety and pain are significant in adults’ trephine bone marrow biopsies.

Several studies on the clinical effectiveness of trephine biopsy were recently published. However, the findings of these investigations were never reported in a survey based on anxiety, pain, needle type, time to complete biopsy, needle bending, frequent recurrences, bone texture, and other characteristics. The primary objective of this study has been to assess numerous aspects of the trephine BMB method, viz., anxiety, pain, needle type, time to finish biopsy, needle bending, frequent recurrences, bone texture, and other factors. The aim was to comprehensively understand the process and its possible use in patient care, consequently increasing awareness of the trephine biopsy’s effectiveness. The secondary objective has been to evaluate the difficulty of the trephine bone marrow biopsy procedure for bone marrow inspection, the level of pain and anxiety experienced by patients, the frequency of needle bending during the procedure, the number of repetitions required, and the precision of sample collection. In addition, the outcomes include evaluating the frequency with which adequate histologic specimens have been obtained and keeping track of the number of needles that passed or bent throughout the procedure. These objectives aimed to provide valuable insights into the challenges and outcomes associated with the trephine BMB procedure, contributing to improved effectiveness and patient care.

## Materials and methods

The current survey was carried out in compliance with systematic survey and research articles standards. The whole analysis was carried out according to the declaration of Helsinki at PGIMER, Chandigarh.

### Study design and data collection

**Fig. 1:**
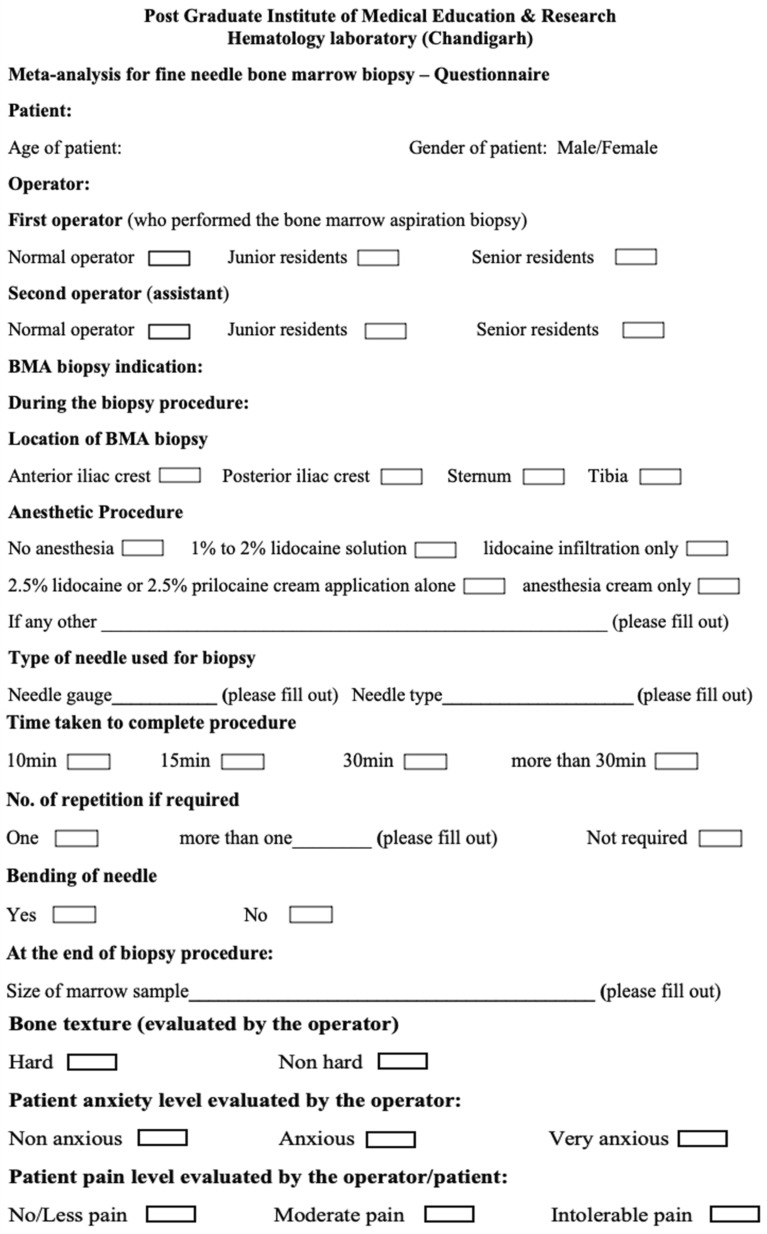
Questionnaire on trephine bone marrow biopsy

The current survey was conducted between March and April 2022 on patients undergoing trephine bone marrow biopsy at PGIMER Chandigarh. PGIMER is a large tertiary care hospital catering to patients from various parts of North India, having numerous departments, including the Department of Clinical Hematology & Medical Oncology (adult), Pediatric Hemato-Oncology unit in the Department of Pediatrics, and Department of Hematology (laboratory-based). The patients included were outdoor and indoor patients undergoing bone marrow procedures for hematological or non-hematological indications. The trephine bone marrow biopsy procedure was performed by trained residents of the Department of Hematology, having experience of 1–6 years. Patients with a language barrier, syncope, and severe psychiatric disorder were excluded from the present study. At the same time, during the procedure, a standardized questionnaire, as shown in Fig. 1, was systematically filled out by the authors. The questionnaire contains 14 inquiries related to the personal experience of the patient and operator before, during, and after the biopsy. The information recorded before and during the procedure includes (refer to Fig. 1): patient information (age, gender), trephine biopsy indication, biopsy location, anesthetic procedure, needle types, and needle bending (if any). The operator was asked to assess the patient’s bone texture and pain level upon procedure completion. Following this, a 1–5-min pressure was employed on the biopsy site to achieve hemostasis. For additional safety, patients were instructed to lie on their back in a prostrate position for additional 10–15 min to apply more prolonged pressure.

### Literature search strategy and data extraction

**Fig. 2:**
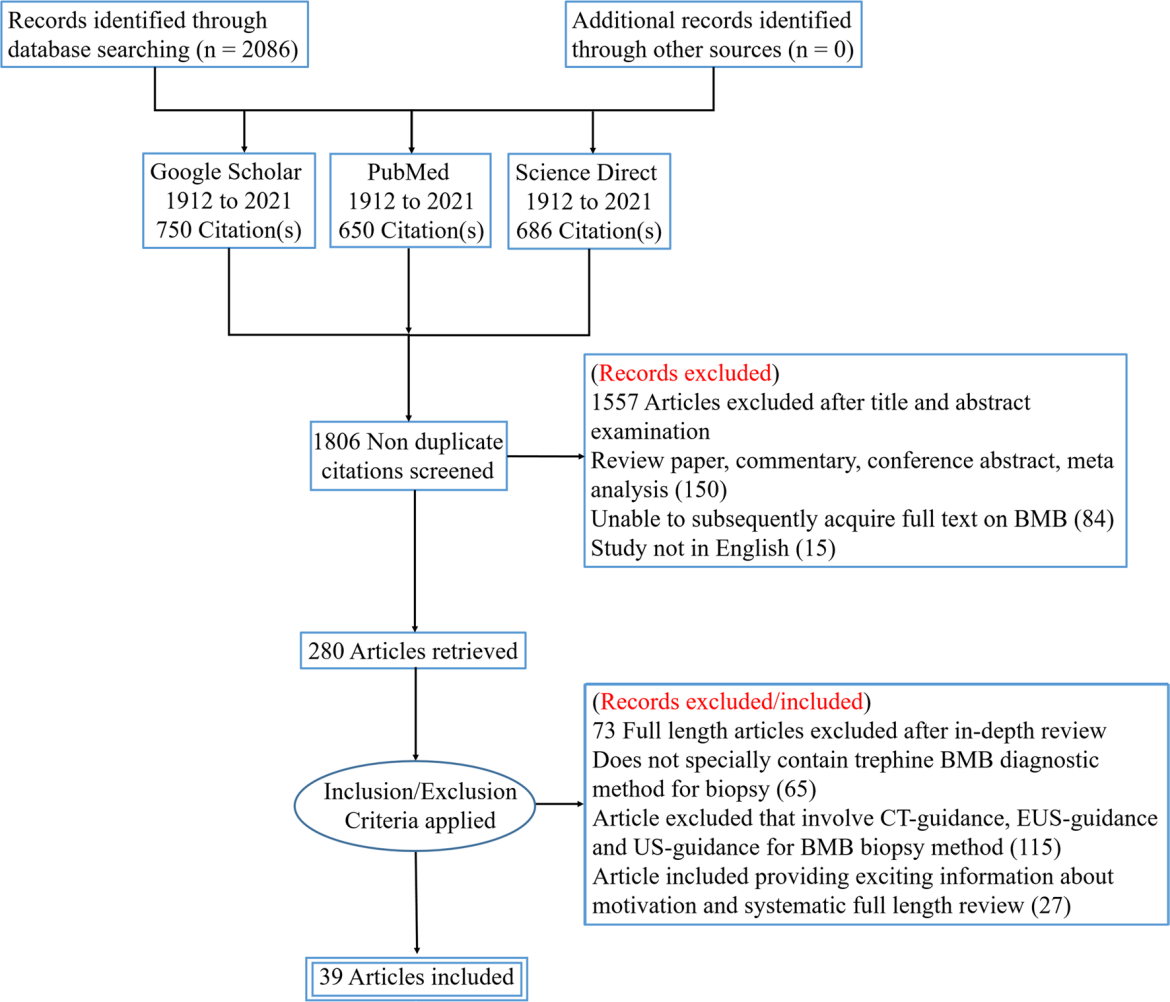
PRISMA flow chart of trephine BMB included studies

To perform this survey, we searched Science direct, MEDLINE, Google Scholar, Research Gate, and PubMed on July 23, 2021, to collect relevant research articles up to 2021. The data for the current analysis were identified without language or time restriction. The search engines were checked by employing various terms, such as: “Bone marrow” AND (“BMA” or “BMB”) AND (“fine needle aspiration” or “trephine biopsy”) AND (“pain” or “anxiety”) AND “BMA and biopsy site.” The research articles were chosen for full-text study based on their abstracts and title. The bibliographies of some research publications were manually searched to enhance the yield of related studies. The data extracted from each incorporated article were the year of publication, study area, author, number of cases (patients), age, gender, sampling type, pain, needle size, suction method, repetitions required, complications, etc. The search engines were used to identify 2086 research articles; 1806 were screened for non-duplication citations. In addition, 84 and 1557 articles were excluded based on the full-text review and information in the abstract and title, respectively, as shown in Fig. 2. Eight research articles were added after studying references from related articles. Finally, 39 articles met all inclusion criteria for the survey concerning the accuracy of diagnosis of BMA and biopsy (Fig. 2).

### Patient population

**Fig. 3:**
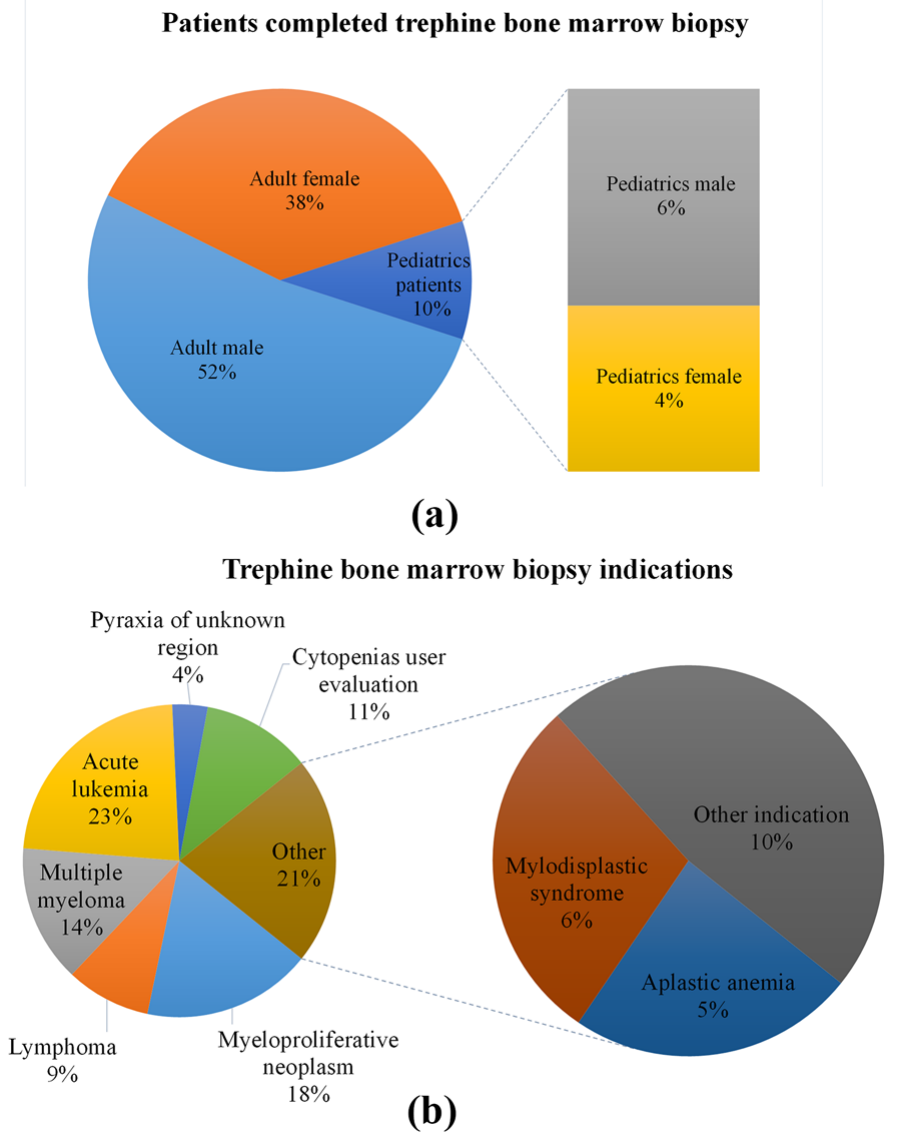
Chart representing **a** patients who completed BMB and **b** indications to perform trephine biopsy

Three hundred questionnaires were filled out by the authors, including 157 (52.3%) adult male patients, 113 (37.6%) adult female patients, and 30 (10%) pediatric patients. The age of patients ranged from 2 months to 85 years (median age of 25 years). Major indications of trephine bone marrow biopsy were 39 (14.23%) patients with suspected multiple myeloma, 63 (22.99%) patients with suspected acute leukemia, 48 (17.52%) patients with suspected myeloproliferative neoplasm, 24 (8.75%) patients with lymphoma for staging and 28 (10.21%) patients with other bone marrow indications. The number of repetitions required was documented and reported in the questionnaire if a patient was subjected to more than one pass during the biopsy procedure. Figure 3a shows the number of patients (*n* = 274) who completed the trephine BMB with respective biopsy process notes and pathology reports. Similarly, Fig. 3b depicts different indications that led the patient to undergo the trephine bone marrow biopsy.

### Trephine bone marrow biopsy needles

**Fig. 4:**
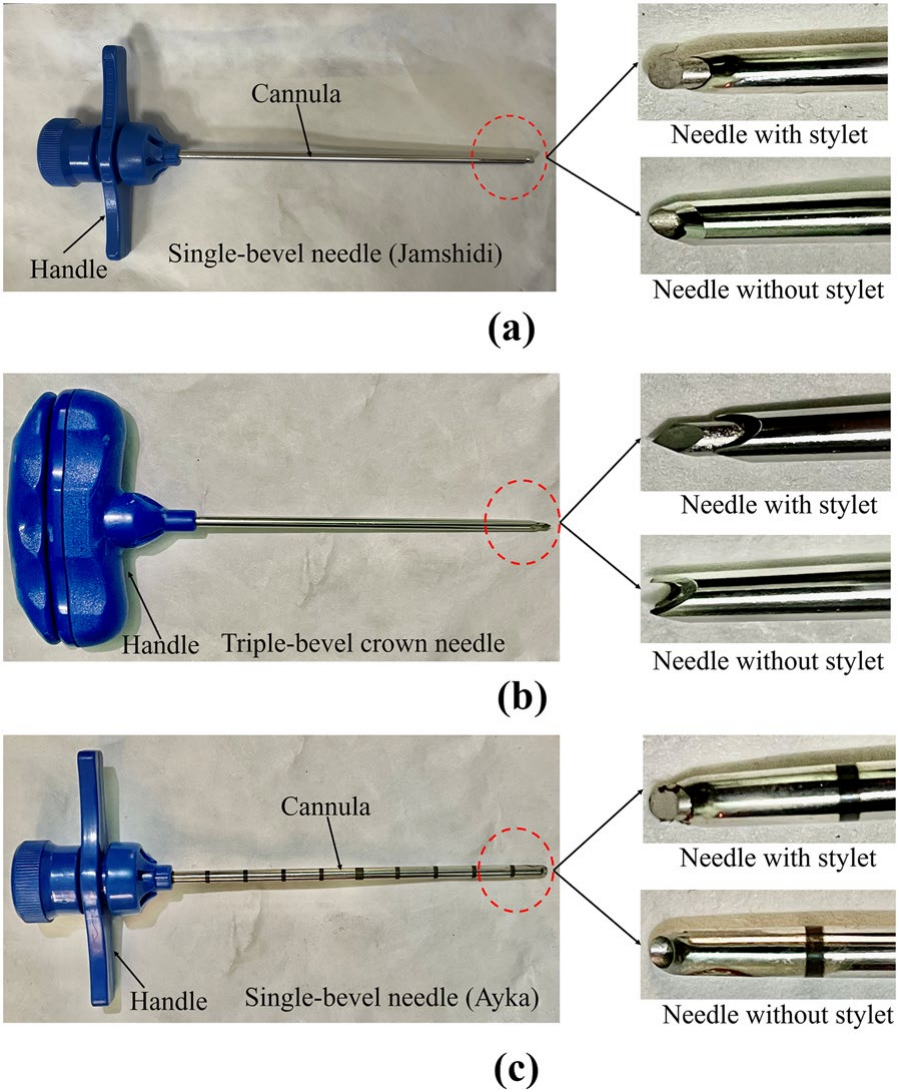
Pictorial view of **a** single-bevel adult biopsy needle, **b** triple-bevel crown tip, and **c** single-bevel pediatrics trephine bone marrow biopsy needle displaying the differences in handles, bevels, and stylets

In the current investigation, two distinct biopsy needle types, single bevel and triple bevel, were used to smooth the trephine BMB procedure. The single bevel trephine biopsy needle was utilized in 264 biopsy cases, while a triple bevel trephine biopsy needle was utilized in 10 biopsy cases. As illustrated in Fig. 4, the triple bevel biopsy needle has a two-piece T handle with procurement cradle, a triple crowned cannula tip with a trocar-tapered sharp-pointed stylet, and three bevels and six sharp cutting edges. A single bevel biopsy needle, on the other hand, has an elliptical stylet, a smooth handle, and a single bevel needle tip with two cutting edges (refer to Fig. 4a, c). For adults, the trephine needle had a diameter of 11 G and a length of 10 cm. On the other hand, the trephine needle used was 13 G and 9 cm long for pediatric patients.

### Typical trephine bone marrow biopsy clinical process

**Fig. 5:**
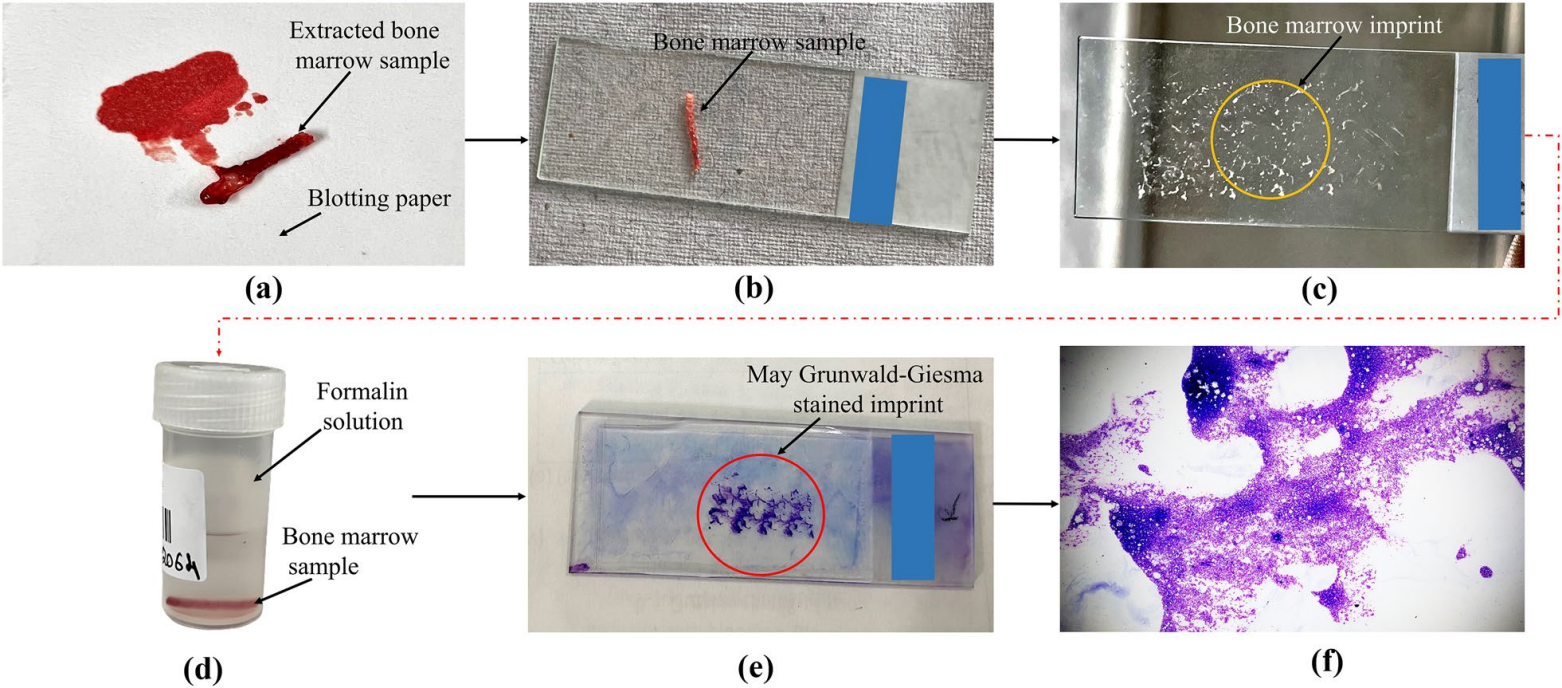
Pictorial representation of pre-and post-processing procedure of trephine biopsy

The comprehensive bone marrow examination was conducted in accordance with PGIMER Chandigarh’s standard operating procedure, and both bone marrow aspirates and trephine biopsies data were collected related to these BMB procedures. Before performing the bone marrow examination, the hematologist briefed the patients regarding the procedure and obtained written informed consent. Patients were requested to lie in a prone position on an examination bed, and specimens from the posterior iliac crest were obtained. Before taking the trephine biopsy, a bone marrow aspiration sample was obtained using a bone marrow aspiration needle (15G/16G for adults, 18G for pediatric patients) and a 20-ml syringe. A hematologist performed the procedure with the assistance of an operator who swiftly prepared bone marrow films and filled various vials (anti-coagulated with ethylene diamine tetra acetic acid (EDTA) and heparin) for further analysis. After the aspiration, the biopsy was performed from the iliac crest using appropriate trephine needles with varying lengths and diameters, as discussed in the previous section. Once the needle was anchored into the bone, the stylet was removed, and the cannula (i.e., outer hollow needle) further penetrated the bone to collect the bone marrow sample. The collected sample can be seen in Fig. 5a. The biopsy needle was withdrawn at the end, and gentle pressure was applied to the biopsy location to minimize excessive bleeding. Following this, biopsy imprint preparations were made from the bone marrow biopsy core. This method involves placing the fresh core biopsy material on a pristine microscope slide (see Fig. 5b). As shown in Fig. 5c, the biopsy sample is carefully placed against a second fresh microscope slide, then the slide is slowly rolled from side to side. It is suggested to produce two or three specimens with various imprints on every plate. Cells on the surface of the biopsy core adhere to the microscope slides when imprint slides are being made. The imprint slides can be stained using the May Grunwald–Giemsa method for morphologic inspection or cytochemical staining and analysis (refer to Fig. 5e, f). Approximately 5–6 ml of 1–2% lidocaine was utilized for local anesthesia, while ketamine and midazolam were given for sedation in children at a dose of 0.1–1 mg/kg. Each patient (or the guardian in the case of children) was informed about the risks of local anesthesia/sedative drugs.

### Quality assessment of trephine biopsy samples

Trephine bone marrow sample quality is mainly characterized as adequate, hemorrhagic, fragmented, subcortical, crushed, and sub-optimal. The length of the biopsy sample collected, a significant determinant of the quality of the sample, is carefully noted after the procedure by hematologists. A quality flaw is also determined (viz., fragmented or crushed); it was observed that such a feature would significantly influence the ability to evaluate from that acquired sample. These factors can be used to assess the quality of a trephine bone marrow sample. For the sample to be classified as ‘crushed,’ more than 20% of the bone marrow must have been destroyed.

**Fig. 6:**
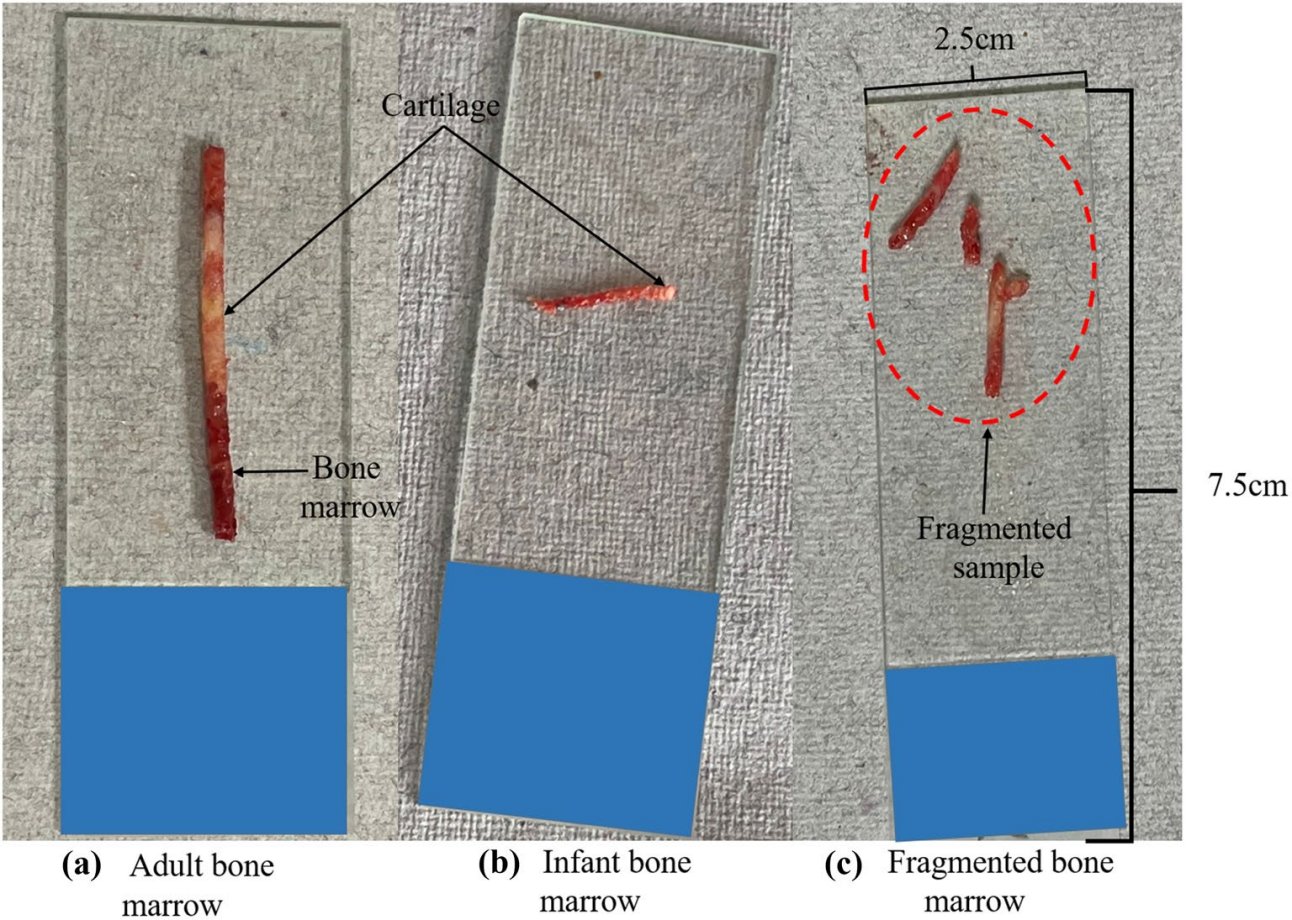
Pictorial representation of **a** adult, **b** infant, and **c** fragmented bone marrow extracted during the trephine biopsy

Furthermore, ‘fragmented’ was defined as having two or more bone marrow pieces primarily segregated without intervening bone or marrow cavity (refer to Fig. 6c). A 4 mm bone marrow or less was classified as ‘small’; however, this quality factor of trephine biopsy was not interrogated. A suitable sample with excellent bone marrow quality parameters was found to determine the diagnosis well. The remaining quality elements imply that the identification of clinical diagnosis was limited or conceptually limited by several circumstances. The crushed material affects bone marrow shape, causing fuzzy nuclei and indefinite cell boundaries, making accurate assessment difficult. Insufficient specimens for pathologic appraisal are suboptimal samples. The subcortical quality factor is a trephine biopsy sample containing mainly cortical bone and very little marrow. Figure 6a, b depicts adult and infant bone marrow samples.

### Statistical analysis and sample size

To address the issue of potential bias, two independent investigators have extracted the data. A comparison of the statistical results and quality scoring has been done, and any discrepancy between the results led to a reanalysis of the original article. Finally, any unresolved issues have been settled by an independent investigator. The results have been presented as mean values with their associated standard deviations. To estimate the precision of the percentage of specimens with evaluable marrow of 4 mm or more, 98% confidence intervals have been considered. These intervals provide a range within which we can be 98% confident that the true population percentage lies. Bivariate logistic regression has been conducted to examine the relationship between each core specimen quality factor and covariates including sex, size of marrow sample, anxiety, pain, bone texture, needle bending, repetition required for the biopsy, indication for trephine biopsy, and length of evaluable marrow.

Sample size refers to the number of observations or replicates that are considered necessary to be included in a statistical study. Based on a sensitivity of 98% in predicting positive core specimen quality factor and taking into account numerous variables related to trephine bone marrow biopsy, a maximum marginal error, also known as precision of estimate, of 2.08% has been chosen. The standard population proportion has been assumed to be 50%. A sample size of 300 patients has to be used to test the general hypothesis. After determining the sample size, it has been determined that a total of 274 patients will be enrolled in the study. This sample size has been deemed appropriate to meet the research objectives and obtain reliable data. Sample sizes are crucial as they represent subsets of a population that are selected for a particular survey or experiment. In the present study, the sample size has been determined considering a finite population, as illustrated in the following equation:

1 $$ n^{\prime} = \frac{n}{{1 + \frac{{z^2 \times \hat{p}\left( {1 - \hat{p}} \right)}}{\varepsilon^2 N}}} $$

where *n* represents the desired sample size from the unlimited population, *ε* is the margin of error, *N* represents the size of the population under consideration, *z* is the *z* score and *p̂* is the estimated population proportion, which is often based on preliminary data or prior knowledge.

## Results

**Fig. 7:**
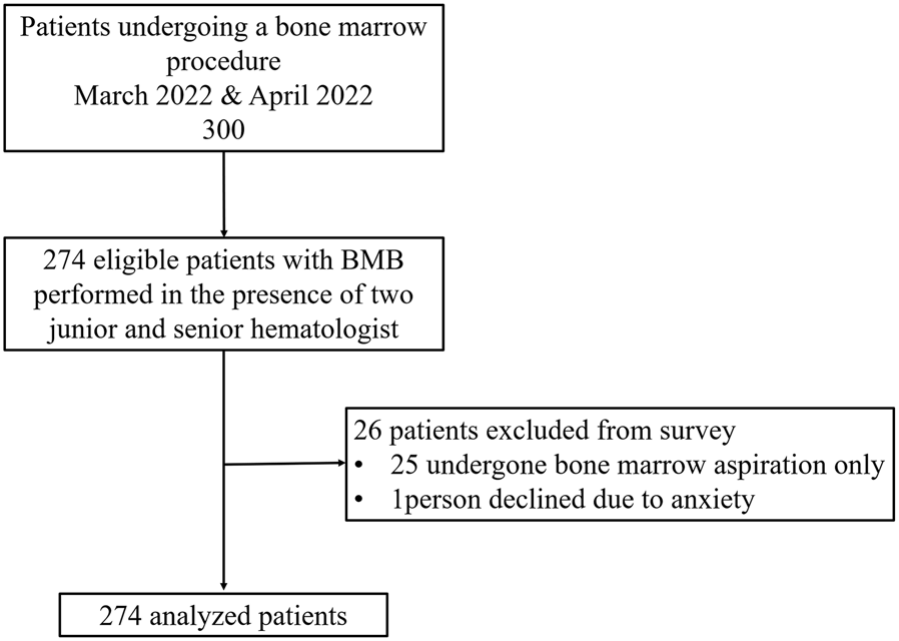
Patients’ analysis flow chart

The present study has considered the data of 300 patients who had undergone bone marrow operations performed at PGIMER between March and April 2022. The research included 274 eligible patients who underwent a BMB in the presence of two senior and junior hematologists (refer to Fig. 7). The entire standardized questionnaire could not be completed for 25 patients, since they underwent BMA only and not BMB. One patient declined to undergo the biopsy technique and has thus been excluded from the study. Finally, 274 patients out of 300 BMB have been studied. With comprehensive biopsy pathology reports, experienced hematologists conducted 274 trephine BMBs from the iliac crest region during the current research period. The age of patients ranged from 2 months to 85 years (174 male and 126 female patients). Of the 274 trephine biopsies, 244 were performed using a single-bevel Care Fusion needle, 20 with a single-bevel Ayka needle, and 10 with a triple-bevel Care Fusion crown needle. The biopsy findings have been examined for bone marrow indication, sample length, clinical indicators, and other laboratory queries. All trephine BMB procedures (*n* = 274, 100%) have been performed under anesthesia. Lidocaine penetration alone (*n* = 244) was the most commonly used anesthetic for the iliac crest bone marrow sample collection in adult BMB procedures. In some instances (*n* = 20), lignocaine prilox cream was given as local anesthesia before the bone marrow procedure to alleviate discomfort. In contrast, ketamine and midazolam alone (*n* = 30) were the most often utilized anesthetics for iliac crest bone marrow sample collection in infants.

**Fig. 8:**
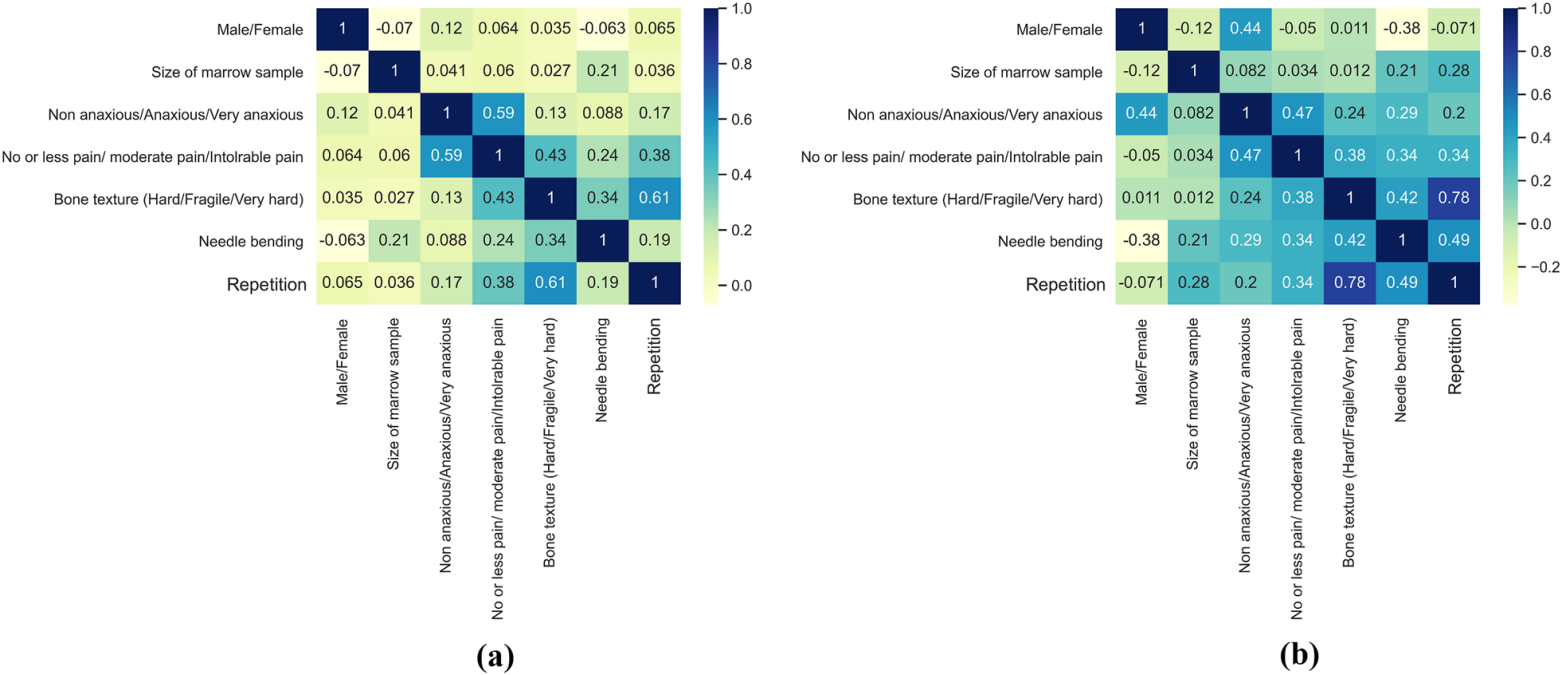
Seaborn heatmap correlation between the variables of trephine bone marrow biopsy

The data collected from the survey are used to examine and summarise the data set’s main features, which are then further represented in a two-dimensional format as a heatmap, as demonstrated in Fig. 8a, b. The correlation data between the output and the input parameters are identified via the seaborn heatmap. The data were, therefore, separated into names and features. The independent variables are sent to the algorithm as features, and the dependent variable is presented as a label. The Seaborn pair graph was used to determine whether the different areas were negatively or positively correlated, and no anomalies were discovered. As depicted in Fig. 8, all the results measured were around − 1 and + 1, indicating that the distribution and correlation of the various fields under consideration are satisfactory. It has been noted that light green color indicates a negative correlation, while purple and bluer colors show a positive correlation between the different features.

### Trephine BMB complications and information quality

**Fig. 9:**
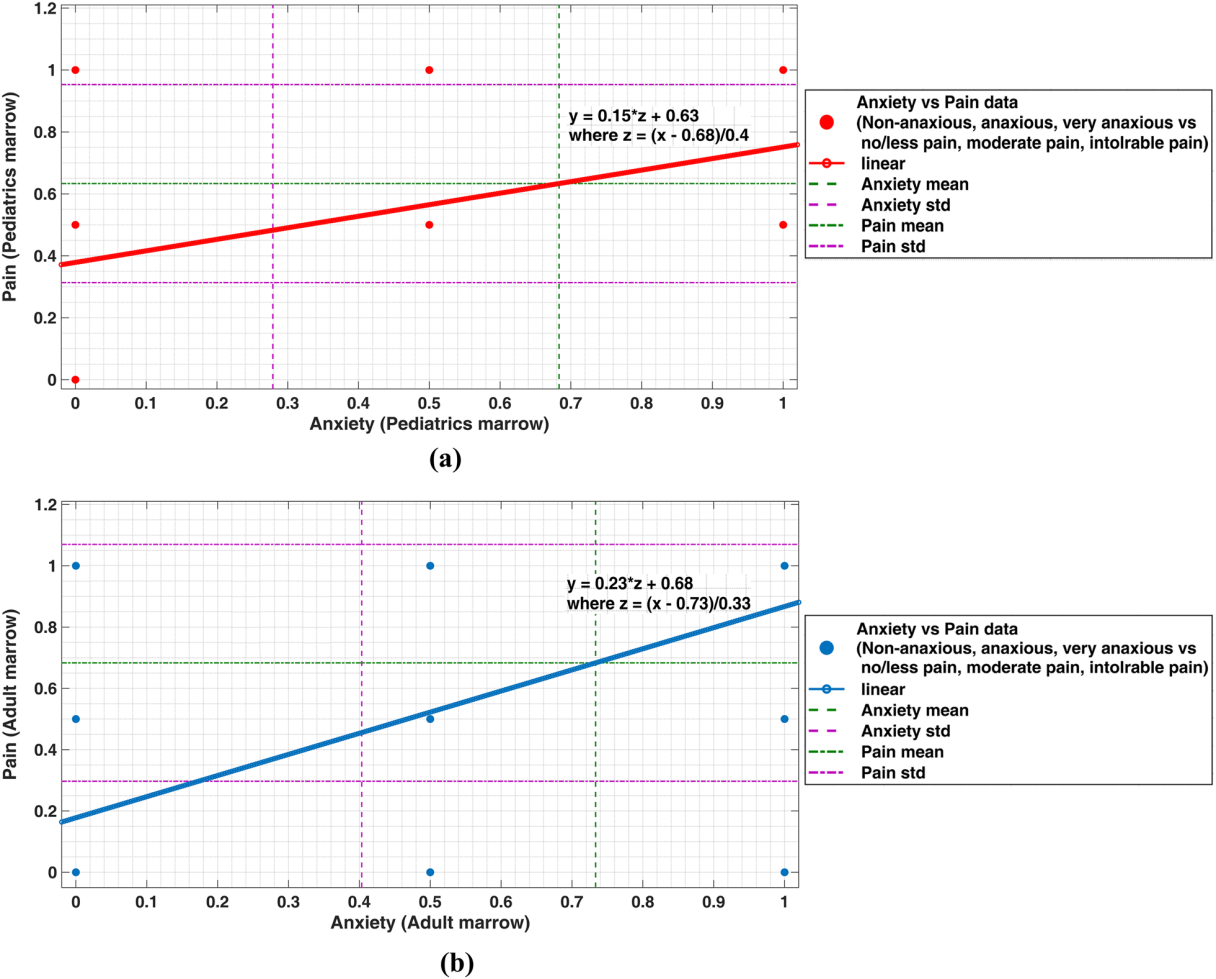
**a** Pediatrics and **b** adult anxiety versus pain level evaluated during the trephine biopsy

Eight incidences of bleeding and dizziness complications were reported during these 2 months of 274 trephine BMB procedures. The most common issues linked with BMB were needle bending, the length of the bone marrow sample, and the number of repetitions needed during the biopsy. The complications associated with trephine BMBs, such as pain, bleeding, dizziness, etc., are shown in Table 1. In the current study, all patients were anxious before and during the procedure, and hence they experienced no/less pain, moderate pain, and intolerable pain during the trephine biopsy procedure (refer to Fig. 9). From these 274, the patients were evaluated by the authors and hematologist as non-anxious for 34 BMB (12.4%), anxious for 92 (33.57%), and very/highly anxious for 148 (54%) BMB cases. It was also observed that the quality of the information provided during the biopsy process could help reduce anxiety to a certain level. The information quality provided before and after the procedure was overall good. In the present study, 232 out of 274 BMB (84.6%) patients believed they were well-informed and satisfied with biopsy-related information before and after the trephine BMB procedures. In 232 inspections, the patients were satisfied with the information provided before, during, and after the procedure; 133 (57.3%) of them became non-anxious, 75 (32.3%) remained anxious, and 24 (10.3%) were highly anxious. In contrast, 42 (15.3%) patients were not satisfied with the information provided before, during, and after the process; of these patients, 4 (9.52%) were non-anxious, 8 (19%) of them were anxious, and 30 (71.4%) were highly anxious (*p* = 0.15). Of the 274 patients who underwent the trephine BMB procedure, only one refused to undergo the BMB procedure due to high anxiety. Providing adequate information regarding the biopsy process might avoid anxiety, and there is no doubt that the trephine biopsy specialist should keep the procedure as brief as possible. Additional complications were revealed as an effect of premedication. Forty-five (16.4%) patients were given premedication before the biopsy procedure, mainly with the appropriate dosage of Prilox Lignocaine cream and Toplap Lidocaine cream. Five (11.1%) experienced the side effects (viz., rashes and reddishness), but no one was critical enough to need extensive hospitalization. Premedication before the procedure helps reduce the pain to some extent but does not help avoid intolerable pain.

**Table 1:** Table showing complications associated with trephine BMB inspections

	Number of patients	Percentage
*Trephine biopsy-related complications*		
Anxiety		
Non-anxious	34	12.4
Anxious	92	33.57
Very/highly anxious	148	54
Pain		
No/less pain	41	14.96
Moderate pain	82	29.92
Intolerable pain	151	55.1
Infection		
Yes	0	0
No	274	100
Bleeding		
Yes	3	1.09
No	271	98.9
Dizziness		
Yes	5	1.82
No	269	98.1
*Premedication-related complications*		
No		
269	98.1	
Rashes		
2	4.5	
Reddishness		
2	4.5	
Fatigue		
1	2.2

### Aspects related to pain

Various issues were covered in the questionnaire surveyed by patients and operators, while some aspects of pain were considered when the procedure was completed. The authors and hematologist evaluated the patients as experiencing no/less pain for 41 BMB (14.96%), moderate pain for 82 (29.92%), and intolerable pain for 151 (55.1%) BMB procedures (refer to Table 1). Out of 274 patients, 251 (91.6%) underwent the biopsy procedure for the first time, while the remaining 23 (8.4%) already had previous bone marrow biopsy procedure experience. From the 251 patients who had their first bone marrow biopsy, 25 (9.9%) were anxious, 86 (34.3%) were non-anxious, and 140 (55.7%) were highly anxious. On the other hand, out of the 23 patients who had already had a previous experience, 9 (39.1%) were anxious, 6 (26%) were non-anxious, and 8 (34.7%) were highly anxious, respectively. Thus, the proportion of patients experiencing high anxiety was less in those who already had an earlier experience with a trephine biopsy procedure. Anxiety was a significant predictor of pain during the procedure, and it was verified that the patient’s psychological condition could affect the existence of pain, as detailed in Fig. 9. It has been reported that most of the previous bad experiences of trephine BMB procedures might cause anxiety and fear for future BMBs and have been linked to intolerable pain [15]. Age does not affect unbearable pain. Fifty-two percent of 213 patients under 60 years of age complained about severe pain. Of the 54 cases over 60 years of age, 51.9% reported intolerable pain during the trephine BMB procedure (*p* = 0.22).

Similarly, in patients under 13 years of age (i.e., pediatric patients), 10% of these 30 patients reported less pain, 53.3% reported moderate pain, and 36.6% reported intolerable pain with sedatives (*p* = 0.43). Ninety-eight (35.7%) trephine BMB inspections were performed by hematologists having more than 250 BMBs of experience. One seventy-six (64.2%) cases had tolerable pain inspected by a clinician with lower than 50 BMB inspection experience (*p* = 0.56). The clinician’s experience did not affect the patient’s anxiety and pain level.

### Bone marrow length and repeat attempts required

From 185 (67.5%) inspections, the trephine BMB was successfully achieved with a single attempt, and a maximum of 4 attempts was required to achieve the appropriate sample. The number of patients having intolerable pain was 34.6% for 185 cases with one attempt for trephine biopsy compared to the highest number of 72.2% for 113 cases with repeated BMB attempts (*p* = 0.68). To evaluate the trephine BMB sample quality, we initially compared bone marrow length in trephine biopsy samples. The triple-bevel needle was related to a smaller length of evaluable bone marrow with a mean variation of 0.13 cm.

**Fig. 10:**
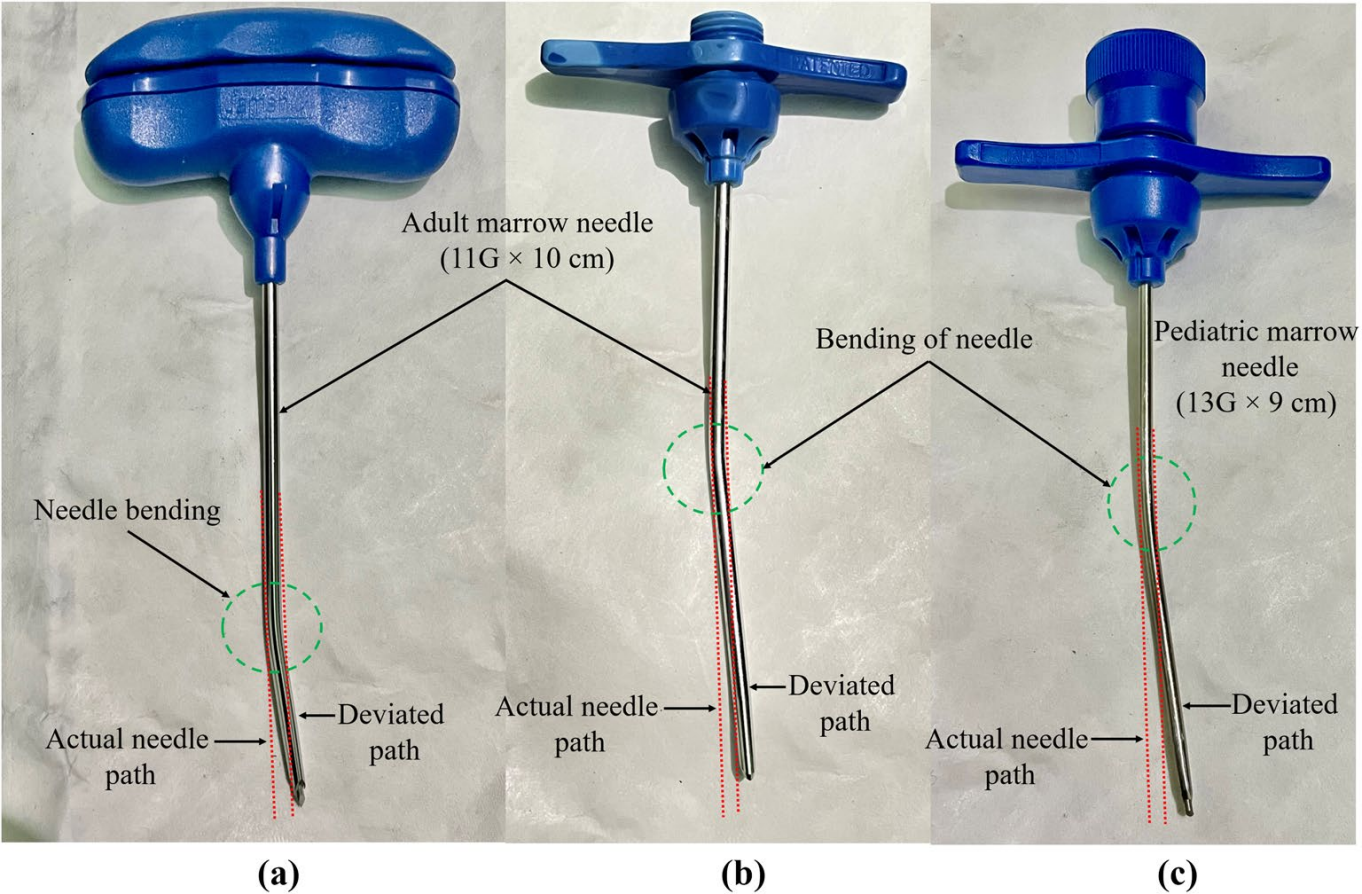
Pictorial representation of different needle types **a** triple bevel needle (adult marrow), **b** single bevel needle (adult marrow), and **c** single bevel needle (pediatric marrow) bending during the biopsy procedure

Moreover, the number of patients having 1.5 cm or more marrow length was significantly less for the case of triple-bevel crown tip (3%; 95% CI 1–5%) needle as compared to single bevel needle (69.3%; 95% CI 62.2–78.8%). The collected information demonstrated that triple-bevel crown tip needles cause less pain than single-bevel needles but are associated with poorer quality BMB samples. However, the number of patients who underwent biopsy using triple bevel crown tip needles was very small (*n* = 10). The number of repetitions and size of bone marrow were also affected by the bone texture of the patients. In the present examination, it has been noticed that the hard (hard bone represents normal bone texture) or very hard bone texture might be related to intolerable pain. Under these conditions, it was experienced that the BMB takes longer to complete, and the bending of the needle is more frequent, as shown in Fig. 10a–c.

**Fig. 11:**
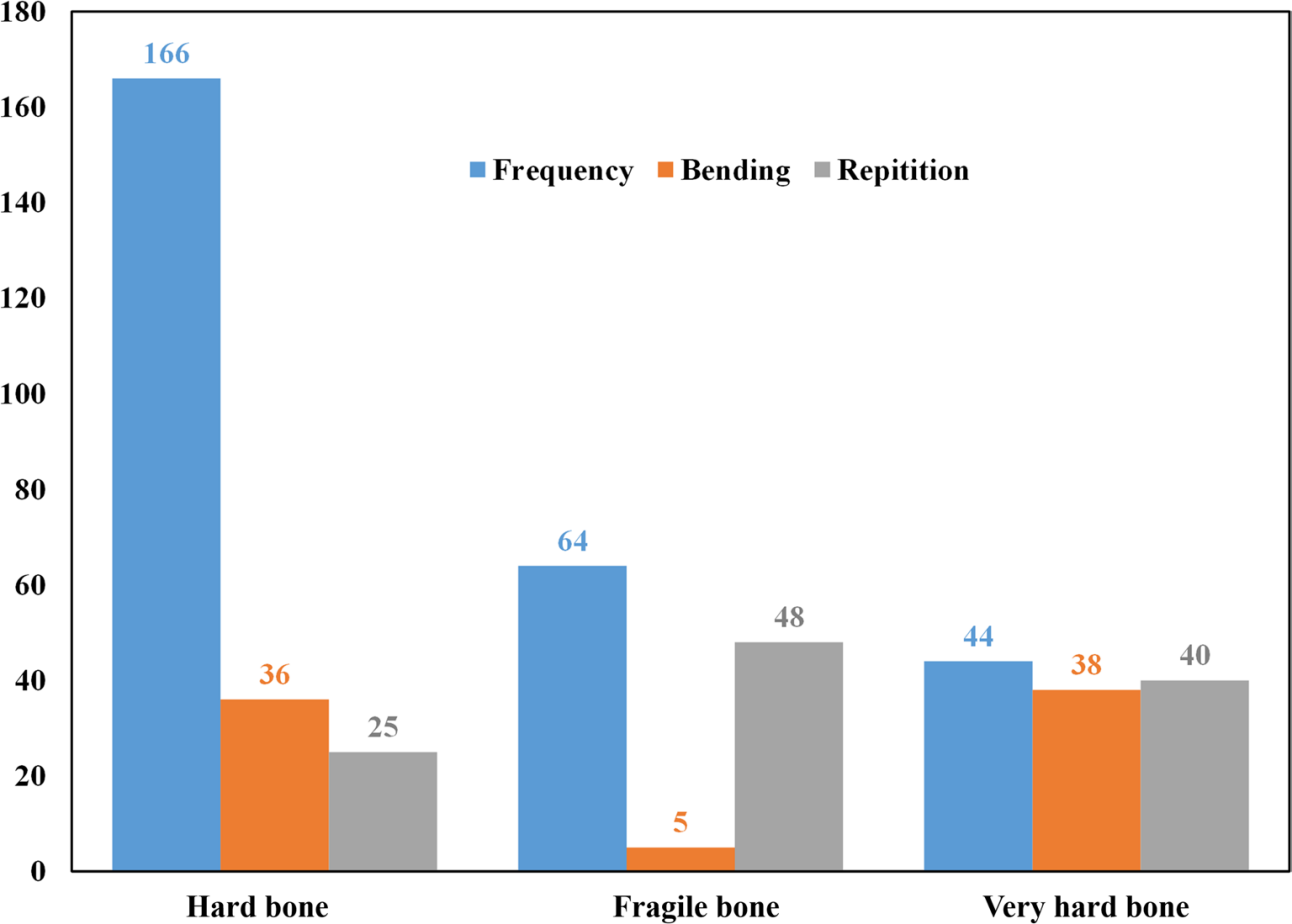
Effect of bone texture on the needle bending and number of repetitions required

Similarly, with fragile bone texture, the bone was very soft, and locating the needle on the iliac crest surface was challenging. Due to the bone’s fragile nature, the bone marrow biopsy length was much shorter than that of the hard bone. Needle bending and the number of repetitions required were significantly affected by the patient’s bone texture. Figure 11 depicts how bone texture affects needle bending and the number of repetitions required during the BMB procedure. Of 274 cases, bone texture for 60.5% of cases was evaluated as hard, 22.3% as fragile, and 16% as very hard (clinical evaluation). Needle bending was recorded in 79 (28.8%) cases out of 274 cases, as shown in Fig. 11. In the ‘very hard’ bone group, needle bending was noted in 86.3% (38 out of 44 cases), while in the hard and fragile group, it was noted in 21.7% (36/166) and 7.8% (5/64), respectively. This indicates that needles bent much more frequently when the bone was assessed as very hard. Needle anchoring is complex and results in frequent needle bending due to the very hard nature of bone. Similarly, during the BMB procedure, 113 cases had to undergo repetitions because of insufficient bone marrow sample acquisition (refer to Fig. 11). This was much more frequent in fragile and very hard bones [48/64 (75%) and 40/44 (90.9%), respectively] compared to hard (normal texture) bones [25/166 (15.1%)]. In the case of fragile bone, the crest boundary layers are soft and often break, making it difficult for the hematologist to anchor the trephine needle in the bone and increase the repetition rate.

**Fig. 12:**
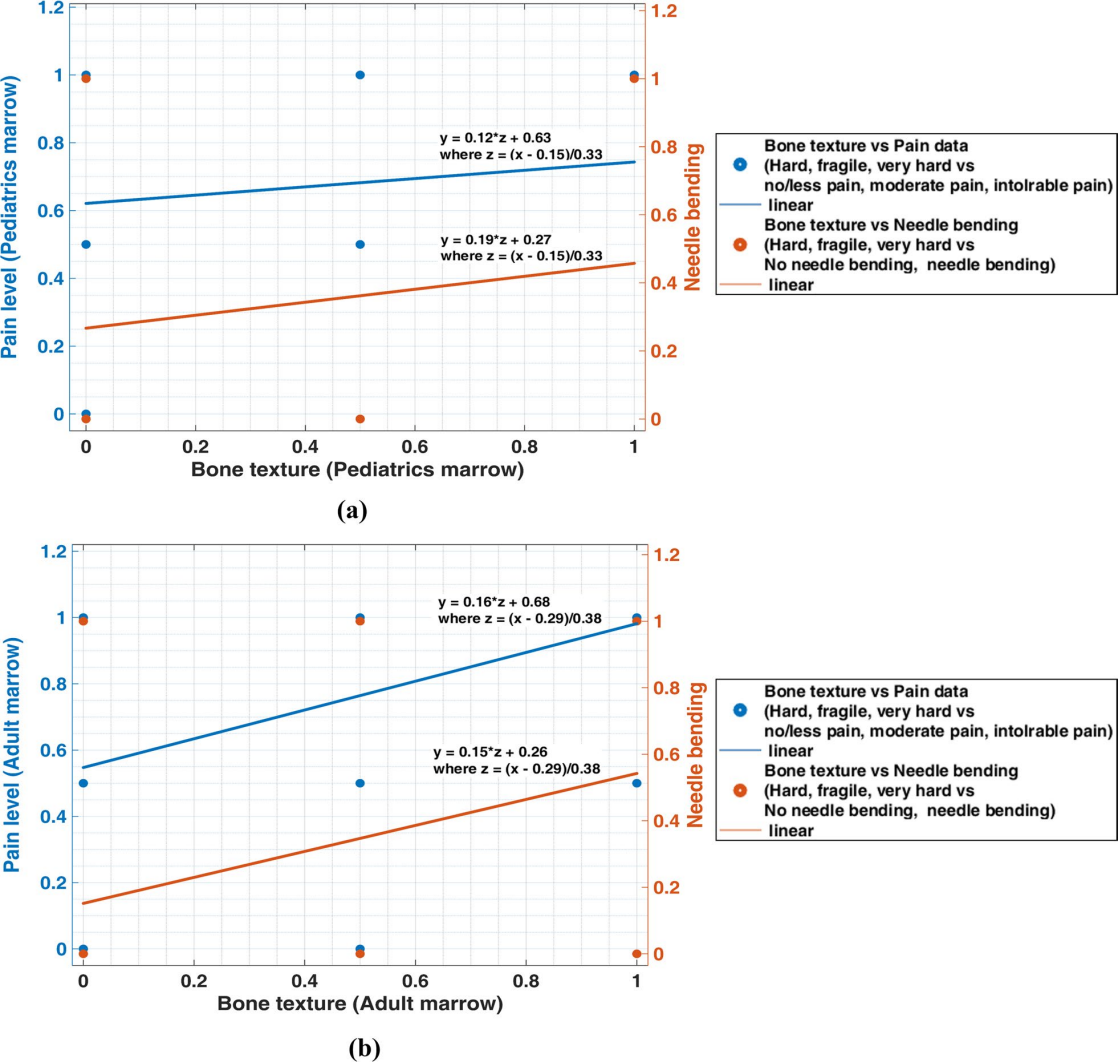
**a** Pediatrics and **b** adult bone texture versus pain level and needle bending evaluated during the trephine biopsy

Figure 12 represents the nature of different bone textures and their effect on pain level and needle bending. Figure 12a, b clearly shows no significant relationship between bone texture and pain. During pediatric and adult trephine biopsy evaluations, patients with a different type of bone texture, i.e., hard, fragile, and very hard reported less moderate and intolerable pain, respectively (refer to Fig. 12). Because of the nature of bone texture, most patients experience intolerable pain during the procedure, as shown in Fig. 12a, b.

### BMB indication-based marrow length

In the present inspection, we also consider various target lengths of bone marrow based on the indication for BMBs. Regarding bone marrow length, depending on indication: 18 BMBs (6.65%) were performed to diagnose suspected myeloproliferative neoplasm with an average evaluable bone marrow length of 2.5 cm. Twenty-four (8.75%) suspected lymphoma, 39 (14.23%) suspected multiple myeloma, and 23 (8.39%) pancytopenia trephine BMB cases were explored with an average bone marrow length of 2.1 cm, 1.6 cm, and 1.9 cm, respectively. Similarly, 23 (8.39%) out of 274 cases with suspected acute leukemia had an average bone marrow length of 1.8 cm, 29 (10.58%) suspected chronic myeloid leukemia and 39 (14.23%) suspected acute myeloid leukemia had an average bone marrow length of 1.9 cm each. More fragmented BMB samples were reported compared to acute and chronic myeloid leukemia in the case of suspected acute myeloid leukemia. The complete evaluable bone marrow length compared with the BMB indication is shown in Table 2. It can be seen from Table 2 that the minimum length of the biopsy sample acquired during the procedure is less than 1.5 cm (1.5 cm gold standard suggested by WHO) for all the biopsy indications. Therefore, an optimum needle design or an external arrangement is required to ensure a minimum biopsy length of 1.5 cm or more. We have encountered 10 (3.64%) bicytopenia and 17 (6.2%) myelodysplastic syndrome indications with an average bone marrow length of 1.8 cm and 2.3 cm.

**Table 2:** Correlation between trephine biopsy indication-based and bone marrow length

Trephine biopsy indications	No. of cases	Percentage (%)	Minimum bone marrow length (cm)	Maximum bone marrow length (cm)	Mean length (cm)	Standard deviation
Myeloproliferative neoplasm	48	17.52	0.5	4	2.12	0.76
Lymphoma	24	8.75	0.6	3.5	2.1	0.77
Multiple myeloma	39	14.23	0.3	3.8	1.59	0.82
Acute leukemia	63	22.99	0.5	3.5	1.72	0.77
Pyrexia of the unknown region	10	3.64	0.5	2.3	1.47	0.67
Cytopenias user evaluation	31	11.31	0.3	4	1.88	0.79
Myelodysplastic syndrome	17	6.20	0.8	4	2.28	0.76
Aplastic anemia	14	5.10	0.2	3.2	1.74	0.89
Other indications	28	10.21	0.2	2.9	1.77	0.69

### Interpretation-based BMB marrow length

The study of bone marrow contains quantitative and qualitative data about marrow cells, as well as morphologic characteristics about every cell line. The consistency of the sample is an essential aspect of interpreting bone marrow samples. Analyzing BMB length in relation to the hematological diagnosis provides valuable insights for diagnostic accuracy and disease categorization. A variety of cellular components in the bone marrow are changed in various hematological diseases. By examining the length of the BMB, clinicians can ensure an adequate representation of the bone marrow microenvironment, enhancing diagnostic accuracy by capturing the characteristic features of each disorder. Furthermore, the length of the BMB can aid in disease categorization and subtyping by determining the scope and severity of the condition. The length of bone marrow based on trephine biopsy interpretation is provided in Table 3. Following the interpretation, acute leukemia was reported to be the most prevalent cancer amongst those who underwent bone marrow examination in our study, with 73 (26.64%) cases diagnosed. According to the interpretation of results, acute leukemia has a minimum and maximum marrow length of 0.2 cm and 4.3 cm, respectively, with a standard deviation of 0.78.

**Table 3:** Table presenting interpretation-based BMB marrow length for different cases

Trephine biopsy interpretation	No. of cases	Percentage (%)	Minimum bone marrow length (cm)	Maximum bone marrow length (cm)	Average length (cm)	Standard deviation
Myeloproliferative neoplasm	39	14.23	0.3	4	2.20	1.0
Lymphoma	23	8.39	0.5	3	1.80	0.76
Multiple myeloma	23	8.39	0.2	3.2	2.0	0.72
Acute leukemia	73	26.64	0.2	4.3	1.93	0.78
Myelodysplastic syndrome	7	2.55	1	2.6	1.51	0.63
Aplastic anemia	16	5.83	0.5	3.1	1.84	0.81
Other interpretation	93	33.94	0.4	4	1.86	0.83

The myeloproliferative neoplasm was the second most prevalent interpretation in the current study, accounting for 39 cases (14.23%), with an average marrow length of 2.20 cm and a standard deviation of 1. Another diagnosis presented as an interpretation in the report following the examination of samples in the current study was aplastic anemia. Of the 274 cases, 16 (5.83%) were identified as aplastic anemia, with a minimum and maximum marrow length of 0.3 cm and 3.1 cm, respectively, and a standard deviation of 8.1. Other cancers diagnosed during this survey were multiple myeloma (8.39%), lymphoma (8.39%), and myelodysplastic syndrome (2.55%). We found 8.39% of multiple myeloma and lymphoma cases, with average bone marrow lengths of 1.80 cm and 2.0 cm, respectively. Myelodysplastic syndrome was diagnosed in only 7 (2.55%) of 274 cases. The minimum and maximum bone marrow length for myelodysplastic syndrome range from 1 to 2.6 cm with a standard deviation of 0.63, as shown in Table 3.

### Surface roughness

**Fig. 13:**
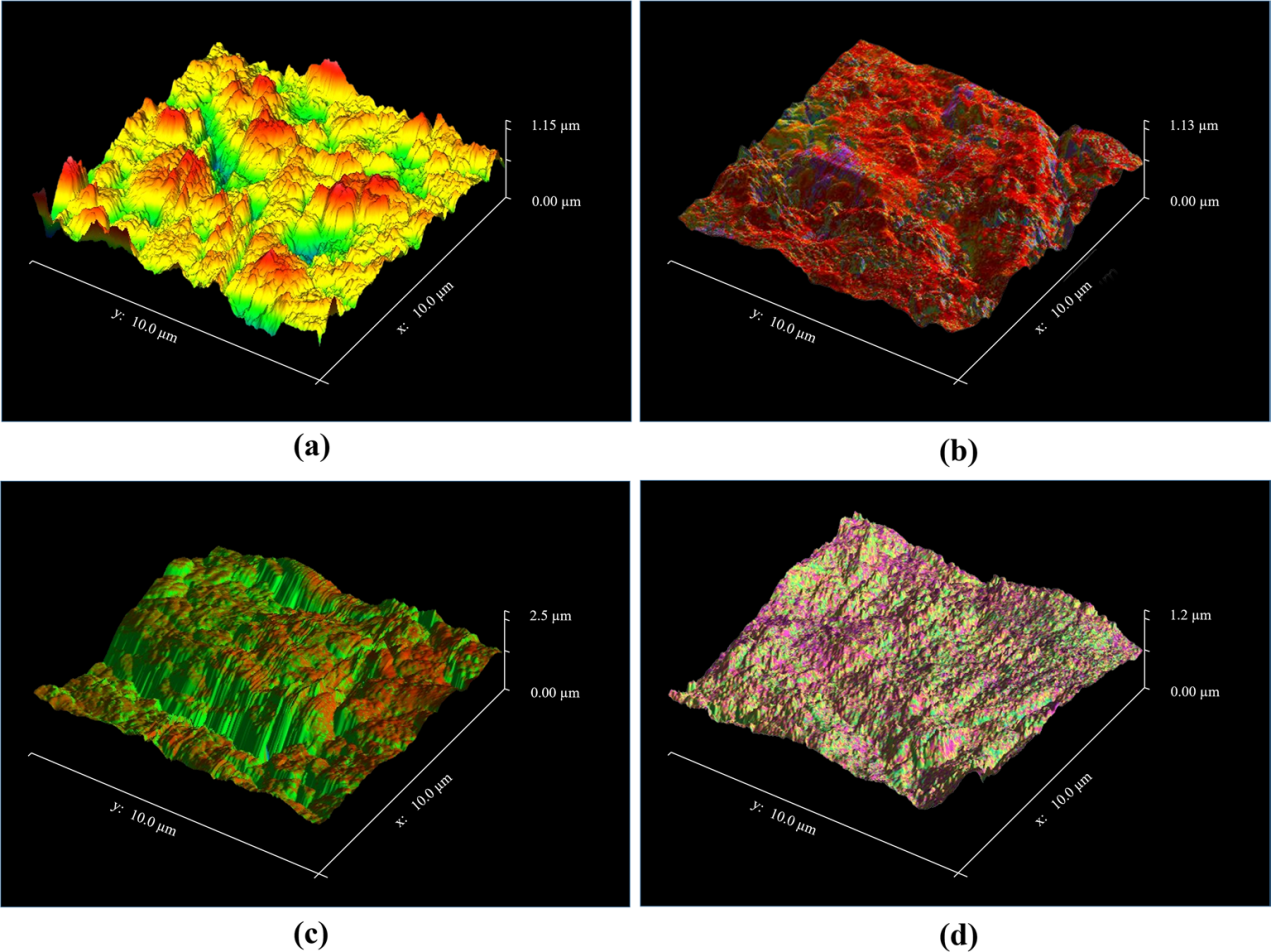
Different needle interior surfaces with typical surface topography: **a** Care Fusion single-bevel, **b** Ayka single-bevel, **c** triple-bevel crown tip, and **d** pediatric BMB needle

The inner needle surface roughness was measured using a Bruker Multimode Atomic Force Microscope with a vertical resolution of 1–5 nm after sectioning the needle to expose the internal surface. Figure 13 depicts microscopic images of the internal surface of an adult marrow biopsy needle (both single and triple-bevel needle) and a pediatric marrow needle. However, the BMB needles cannot be drawn using a mandrel, so their internal surface might be rough. In the present analysis, we found that the usual surface roughness (Ra) for the inner surface of commercial needles was between 1.3 and 3.4 µm. Figures 12d–13a depict the inner surface roughness of Care Fusion single-bevel, Ayka single-bevel, triple-bevel crown tip, and pediatric BMB needle with 0.79 µm, 0.70 µm, 1.96 µm, and 0.64 µm, respectively. As tissue moves beside the wall during operations, its rough internal surface might provide considerable frictional force to the needles. Because of this reliance on tissue length, existing trephine BMB needles are unsatisfactory, since their surface roughness restricts the quantity of tissue that can be collected. The bone marrow sample inside the needle was crushed considerably because of the high surface roughness and inner friction force. As the biopsy core within the roughened needle was crushed more, the protrusion length was more significant, and the contact length was shorter. The needle’s inner frictional force influences the length of the biopsy. The biopsy length increases as the inner friction force decrease. Lowering the inner friction force may reduce the resistance to the biopsy core moving within the needle and lengthen the biopsy. The findings collected from the examination reveal that the triple-bevel crown tip had the shortest biopsy length, because it had higher inner surface roughness and friction force as compared to other needles.

## Discussion

The present study conducted at PGIMER, Chandigarh, is a survey evaluating the pain during the BMB procedure, needle bending, number of attempts, bone texture, etc. The examination assured the safety of the process without any complications related to trephine BMB except pain. Some other complications associated with trephine BMB, such as prolonged pain, infection, fatigue, and nausea, were rare and unreported. The only complications reported were bleeding and dizziness in some cases. Trephine biopsies are essential for diagnosing, prognosticating, and follow-up of various hematological and non-hematological disorders. Various biopsy needles are employed in diagnostic procedures [24, 25]. In this study, we executed a prospective survey evaluating the quality metrics of 274 BMB samples with two different Care Fusion and one Ayka needle design. These needles differed in the number of cutting edges, stylet shape, several bevels, and the shape of the handle and cradle receiving the marrow (refer to Fig. 5). Intolerable pain was reported by 55.1% of the patients during the trephine BMB, higher than the literature data in previous surveys, i.e., 3.7% and 15.9% of patients complained of unbearable pain [15, 26]. This variation in data may be due to the design of the inspection, the various factors for intolerable pain, and the standardized procedure.

On the other hand, compared with previous studies [26–35], the authors observed no significant association between intolerable pain and the use of premedication. In 2004, Giannoutsos et al. [28] revealed that 76 out of 112 patients chose not to take any premedication before the bone marrow examination and that 74 out of 76 patients were self-satisfied with their choice. Degan et al. [15] pointed out that a detailed explanation of the biopsy process in an instructive and comprehensive way permitted building trust among patients and physicians. It was necessary to confirm the patient’s self-confidence level and was integrated with less pain during the iliac crest trephine BMB procedure [15, 16].

The different models of needles evaluated in the present examination had distinctive properties that make them convenient in specific diagnostic procedures. The triple-bevel crown tip needle has auxiliary cutting edges and sharp stylets to engage and penetrate cortical bone. The triple-bevel trephine needles are believed to generate small insertion and extraction forces to puncture cortical bone, as per the Care Fusion brochure. Furthermore, the single-bevel biopsy needle helped retain the BMB sample with considerable lengths of assessed marrow and longer overall core quality. The side effects of the above-described premedication and its mortality and morbidity are usually very few. This may be further significant in outdoor patients, who must return home.

As some BMB indications such as leukemia, lymphoma, and myelodysplastic syndrome need a considerable length of bone marrow to attain satisfactory sensitivity, it must be suggested to deploy the trephine needle, which supports the objective of a more significant length of bone marrow sample [36]. Usually, it is also beneficial to support the trephine BMB needles, which augment the bone marrow sample quality to avoid excessive biopsy repetitions and increase diagnostic yield. Furthermore, the triple-bevel trephine needle utilizes an acquisition cradle for smooth bone marrow sample acquisition. Variations in the needle handle can impact how the clinician interacts with the trephine biopsy needle, as in the case of manual biopsy. These variances may independently or together disrupt the sample quality, where the triple-bevel trephine needle-cutting procedure and specimen acquisition method typically yield a low bone marrow sample. In contrast, triple-bevel needle shear stress permits a lower insertion force that fragments the bone microarchitecture and inherent bone marrow.

The primary outcomes of our examination were the aspects conducted regarding unbearable pain. Neither gender, number of repetitions, premedication, age, nor hematologist experience, or information quality was significantly related during the trephine biopsy. These observations differ from earlier reports, except for the hematologist’s experience, premedication, bone texture, and bone marrow length based on BMB indication. However, age was significant in one of the observations, whereas it appears to play a small part in another observation [26, 37]. The key findings were attributed to providing insufficient information to the patient before and during the procedure. A well-satisfied patient with information can establish a mental state and have lesser unbearable pain. Patients with unbearable pain usually had a poor impression of the bone marrow inspection procedure and the doctor.

The advantage of the present examination is that the hematologists/residents were blinded to a specific type of needle. In addition, the analyses were based on the patient’s gender, age, number of previous biopsies, and BMB indications and included differences from biopsy operators, length of bone marrow, level of anxiety/pain, and bone texture to interpret suitable influence produced in these features. An additional advantage of the present study was that we considered the target length of each bone marrow sample based on BMB indication and interpretation, which remained un-attempted by Brestoff et al. [38] in their study. However, the limitation of the present study was that the hematologist performing the BMB with less experience was blinded to a specific type of needle. Still, the type of needle was not fixed. Due to this, hematologists face problems when handling different types of needles during the procedure. The study was restricted to trephine bone marrow biopsy, and aspiration needle and smearing-related results were not included.

Despite these drawbacks, our analysis indicates that trephine biopsy needle type can impact bone marrow quality. We suggest medical facilities performing BMB procedures evaluate the quality design whenever novel needle types are included. The primary objective of these head-to-head evaluations should be to confirm that BMB sample quality and length are not inadvertently compromised. Although the triple-bevel trephine needle employed in the present examination was related to poorer bone marrow quality, there was less pain during the procedure than the single-bevel needle. This needle design may be suitable in some medical situations, such as a patient having dense cortical bone and operators who have not been able to acquire an acceptable biopsy sample with an alternative needle design. Specifying our results, we suggested that adult BMB performed in PGIMER utilize a single-bevel biopsy needle as the preferred technique to attain a BMB sample from the posterior iliac crest. However, if a patient wishes to use another needle design for biopsy or a single-bevel biopsy needle fails to acquire a suitable sample, a triple-bevel trephine needle should be tried.

Bone marrow examination is an effective and adequate technique for diagnosing hematological and non-hematological conditions. A trephine BMB reflects the topographical order of hematopoietic cells inside the marrow structure and provides a descriptive overview of marrow cellularity. The data illustrate a considerable effect of noble information provided via the hematologists/residents to minimize the anxiety/pain during the minimal invasive inspection. This information is frequently undervalued in a routine medical procedure, but it can be beneficial in minimizing the pain during a biopsy procedure. The information also recommends that the benefits and drawbacks of premedication be discussed with the patient, and then that approach should be incorporated into the patient’s selection process.

## Conclusion

This study suggests that patients should be well-informed and be made comfortable during trephine BMB procedure to minimize pain. The posterior iliac crest has been recommended for BMB procedure due to its larger marrow capacity. Local anaesthesia with lidocaine or sedative drugs increased patient tolerance. Pain has been the major complication in both adult and pediatric patients, with 41 (14.96%) reporting no or minimal pain, 82 (29.92%) experiencing moderate pain, and 151 (55.1%) reporting intolerable pain. Premedication reduced anxiety in some patients, although complications have been observed in 5 (11.1%) of the 45 (16.4%) who received it. Bone texture affected the length of the bone marrow sample, with “fragility” and “very hard” texture resulting in shorter samples. “Very hard” bones also had more frequent needle bending and required more repetitions. Modern improvements to trephine biopsy needles with a triple-bevel needle to acquire the bone marrow sample are expected to decrease the pain during the procedure.

## Data Availability

Not applicable.

## Abbreviations

BMA: Bone marrow aspiration
BMB: Bone marrow biopsy
EDTA: Ethylene diamine tetra acetic acid
PGIMER: Post Graduate Institute of Medical Education & Research
